# Combinatorial Host-Response Biomarker Signature (BV Score) and Its Subanalytes TRAIL, IP-10, and C-Reactive Protein in Children With *Mycoplasma pneumoniae* Community-Acquired Pneumonia

**DOI:** 10.1093/infdis/jiad573

**Published:** 2023-12-13

**Authors:** Cihan Papan, Semjon Sidorov, Beat Greiter, Nina Bühler, Christoph Berger, Sören L Becker, Patrick M Meyer Sauteur

**Affiliations:** Center for Infectious Diseases, Institute of Medical Microbiology and Hygiene, Homburg, Saarland University, Germany; Institute for Hygiene and Public Health, University Hospital Bonn, Bonn, Germany; Division of Infectious Diseases and Hospital Epidemiology, University Children's Hospital Zurich, Zurich, Switzerland; Division of Infectious Diseases and Hospital Epidemiology, University Children's Hospital Zurich, Zurich, Switzerland; Center for Infectious Diseases, Institute of Medical Microbiology and Hygiene, Homburg, Saarland University, Germany; Division of Infectious Diseases and Hospital Epidemiology, University Children's Hospital Zurich, Zurich, Switzerland; Center for Infectious Diseases, Institute of Medical Microbiology and Hygiene, Homburg, Saarland University, Germany; Division of Infectious Diseases and Hospital Epidemiology, University Children's Hospital Zurich, Zurich, Switzerland

**Keywords:** *Mycoplasma pneumoniae*, community-acquired pneumonia, TRAIL, IP-10, host response biomarkers

## Abstract

**Background:**

Host-response biomarkers to differentiate bacterial from viral etiology in children with respiratory infections have shown high accuracies, but are understudied in *Mycoplasma pneumoniae* (*Mp*) infections.

**Methods:**

We compared BV scores (0–34 indicating viral etiology, and 66–100 indicating bacterial etiology), tumor necrosis factor–related apoptosis-inducing ligand (TRAIL; pg/mL), interferon-γ inducible protein 10 (IP-10; pg/mL), and C-reactive protein (CRP; mg/L) serum levels between *Mp*-positive (*Mp*^+^) and *Mp*-negative (*Mp*^−^*)* community-acquired pneumonia (CAP) patients. We performed receiver operating characteristic (ROC) curve analyses for clinical features and biomarkers.

**Results:**

Of 80 CAP patients (median age, 6.3 years; 57.5% male), 26 had *Mp*^+^CAP. In *Mp*^+^CAP patients, compared to *Mp*^−^CAP patients, BV scores were lower (14.0 [3.0–27.8] vs 54.0 [12.0–84.8]; *P* = .0008), TRAIL levels were higher (86.5 [67.4–123.0] vs 65.5 [42.5–103.9]; *P* = .025), CRP levels were lower (12.9 [4.0–22.3] vs 36.7 [13.0–132.8]; *P* = .0019), and IP-10 levels were comparable (366.0 [150.2–603.8] vs 331.0 [154.3–878.8]; *P* = .73) (all median [interquartile range]). ROC analyses yielded a comparable discriminatory accuracy for the combination of age, fever duration, and duration of respiratory symptoms, with either procalcitonin or BV score (area under the ROC curve, 0.87 vs 0.86; *P* = .94).

**Conclusions:**

Children with *Mp*^+^CAP have atypically low, viral levels of the BV score, underscoring the complementary role of microbiological testing.

## Teaser

In this study on the host-response biomarker BV score and its subanalytes TRAIL, IP-10, and CRP, children with *Mycoplasma pneumoniae* community-acquired pneumonia have atypically low levels of the BV score, resembling a viral infection.

Novel host-response biomarkers including combination scores to differentiate between bacterial and viral etiology in children with respiratory tract infections have previously shown high diagnostic accuracies using clinician adjudication as a reference standard [1, 2]. However, the combinatorial host-response biomarker signature “BV score” and its subanalytes tumor necrosis factor–related apoptosis-inducing ligand (TRAIL), interferon-γ inducible protein 10 (IP-10, also known as CXCL10), and C-reactive protein (CRP) have been understudied in infections with *Mycoplasma pneumoniae* (*Mp*), a major bacterial cause of community-acquired pneumonia (CAP) in children [3]. Definitive diagnosis of CAP caused by *Mp* can be cumbersome due to the difficulty in differentiating infection from carriage by current diagnostic test methods, including polymerase chain reaction (PCR) and serology [4]. We recently demonstrated in a prospective cohort study of CAP in children that the measurement of specific peripheral blood immunoglobulin M (IgM) antibody-secreting cells (ASCs) by enzyme-linked immunospot (ELISpot) assay improves diagnosis of *Mp* infection in CAP [5, 6]. This test differentiated between *Mp* infection and carriage. Using this new diagnostic test, we were able to identify biomarkers and clinical features associated with *Mp* CAP [3, 7].

Given the relative novelty of the BV score and its promising results in children with respiratory tract infections [1], we decided to evaluate the BV score and its subanalytes in *Mp* CAP using this well-diagnosed cohort. Based on the distinctive microbiological characteristics of the atypical bacterium *Mp* [8], we hypothesized that levels of the BV score and its subanalytes in *Mp*-positive CAP patients differ from the levels found in patients with *Mp*-negative CAP.

## METHODS

We performed a post hoc analysis of the previously published cohort of pediatric CAP patients and healthy controls (HCs), where all enrolled patients were investigated for *Mp* in pharyngeal swab samples by specific real-time PCR [5, 6]. In this cohort, *Mp* PCR-positive results were confirmed with the measurement of *Mp*-specific IgM ASCs by ELISpot assay to differentiate *Mp*-infected patients from carriers with CAP caused by other pathogens or healthy carriers [3]. Pharyngeal swab samples were additionally tested for *Streptococcus pneumoniae* (*Sp*) by real-time PCR, knowing that detection of *Sp* in the upper respiratory tract (URT) is likely colonization and not infection, as coinfection with *Sp* and *Mp* is uncommon whereas co-colonization may be more common [3]. Another 23 viral and bacterial respiratory pathogens were tested using the ePlex respiratory pathogen panel (GenMark Diagnostics, Carlsbad, California) [3]. The cohort was additionally tested for procalcitonin (PCT) levels, as previously reported [3].

We analyzed BV scores (ranging from 0 to 100, according to manufacturer instructions) and the serum levels of the subanalytes TRAIL (pg/mL), IP-10 (pg/mL), and CRP (mg/L). Measurements were performed using Liaison MeMed BV on a Liaison XL (DiaSorin, Saluggia, Italy), as previously described [1].

Biomarker levels were compared between groups by Mann–Whitney *U* test or Fisher exact test, as appropriate. We performed receiver operating characteristic (ROC) curve analyses and calculated the area under the ROC curve (AUC) for the combination of demographic and clinical features (age, duration of fever, duration of respiratory symptoms) with and without PCT, as previously reported [3], or BV. To this end, we took an AUC threshold of ≥0.75 as a marker of adequate discrimination [9]. We compared differential AUCs by utilizing the DeLong test. We performed Spearman rank correlation to test for an association between biomarker levels and *Mp* bacterial loads in genome equivalents (gEq/mL). All reported *P* values are 2-tailed with statistical significance defined as *P* value <.05. Statistical computing and visualization were conducted in R version 4.3.1 software.

## RESULTS

### Study Population

Of 80 CAP patients (median age, 6.3 years [interquartile range {IQR}, 4.4–9.8 years; 57.5% male; 53.8% hospitalized), 26 were *Mp*-positive (*Mp*^+^CAP), determined by detection of *Mp*-specific IgM ASCs; among 25 HCs, 5 were *Mp* PCR-positive (*Mp*^+^HC, carriers) (Supplementary Table 1). In general, *Mp*^+^CAP patients were older than *Mp* PCR-negative CAP (*Mp*^−^CAP) patients (9.2 vs 5.6 years; *P* = .0003) and were less likely to be hospitalized (30.8% vs 64.8%; *P* = .0078) (Table 1).

**Table 1.** Patient Characteristics and Group Comparison Between Patients With *Mycoplasma pneumoniae*–Positive or –Negative Community-Acquired Pneumonia

Parameter	*Mp* ^+^CAP (n = 26)	*Mp* ^−^CAP (n = 54)	*P* Value
Age, y, median (IQR)	9.2 (7.6–11.3)	5.6 (4.2–7.8)	.0003
Female sex	12 (46.2%)	22 (40.7%)	.81
Hospitalization	8 (30.8%)	35 (64.8%)	.0078
Oxygen supplementation	3 (11.5%)	10 (18.5%)	.53
TRAIL, pg/mL, median (IQR)	86.5 (67.4–123.0)	65.5 (42.5–103.9)	.025
IP-10, pg/mL, median (IQR)	366.0 (150.2–603.8)	331.0 (154.3–878.8)	.73
CRP, mg/dL, median (IQR)	12.9 (4.0–22.3)	36.7 (13.0–132.8)	.0019
BV score, median (IQR)	14 (3–27.8)	53 (12–84.8)	.0008
Viral detection	7 (26.9%)	5 (9.3%)	.05
Rhinovirus/enterovirus	3 (11.5%)	2 (3.7%)	.32
RSV	0	2 (3.7%)	1.00
Influenza virus	0	1 (1.9%)	1.00
Adenovirus	3 (11.5%)	1 (1.9%)	.098
Parainfluenza virus	0	1 (1.9%)	1.00
Human bocavirus	1 (3.8%)	0	.33
Bacterial detection	16 (61.5%)	15 (27.8%)	.0065
*Streptococcus pneumoniae*	16 (61.5%)	15 (27.8%)	.0065
*Chlamydia pneumoniae*	0	1 (1.9%)	1.00

Data are presented as No. (%) unless otherwise indicated.

Abbreviations: CRP, C-reactive protein; IP-10, interferon-γ inducible protein 10; IQR, interquartile range; *Mp*^–^CAP, *Mycoplasma pneumoniae*–negative community-acquired pneumonia; *Mp*^+^CAP, *Mycoplasma pneumoniae*–positive community-acquired pneumonia; RSV, respiratory syncytial virus; TRAIL, tumor necrosis factor–related apoptosis-inducing ligand.

### BV Scores and Serum Levels of the Subanalytes

**Figure 1.**
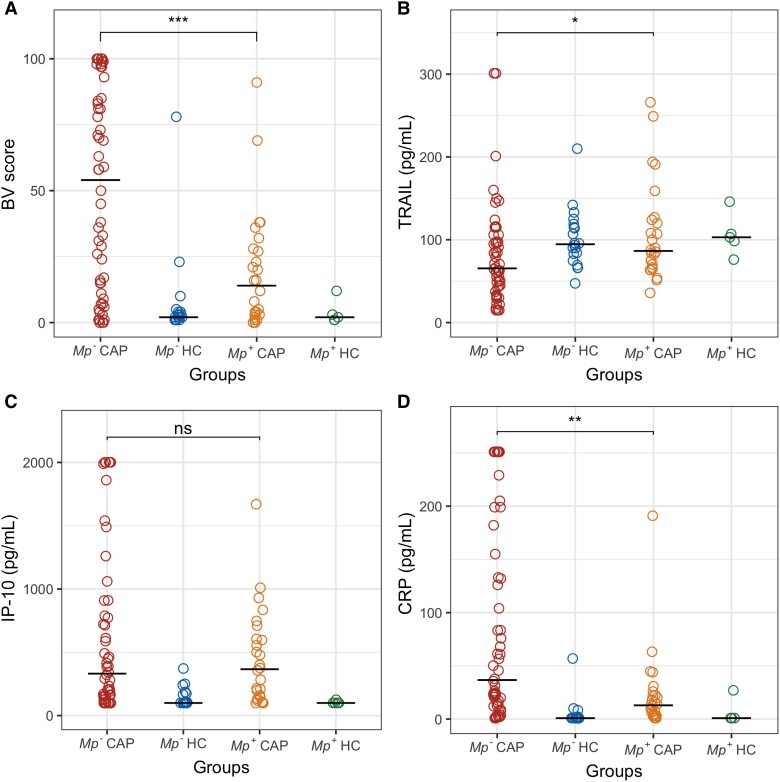
Distribution of BV scores (*A*) and serum levels of the subanalytes tumor necrosis factor–related apoptosis-inducing ligand (TRAIL; *B*), interferon-γ inducible protein 10 (IP-10, also known as CXCL10; *C*), and C-reactive protein (CRP; *D*) among *Mycoplasma pneumoniae*–positive (*Mp*^+^) community-acquired pneumonia (CAP) patients, *Mycoplasma pneumoniae*–negative (*Mp*^−^) CAP patients, *Mp*^+^ healthy controls (HC), and *Mp*^−^HC. *P < .05; **P < .001; ***P < .0001; ns, not significant. BV indicates the host-response biomarker signature composed of TRAIL, IP-10, and CRP.

In *Mp*^+^CAP patients, compared to *Mp*^−^CAP patients, BV scores were lower (median, 14.0 [IQR, 3.0–27.8] vs 54.0 [IQR, 12.0–84.8]; *P* = .0008), while TRAIL levels were higher (median, 86.5 [IQR, 67.4–123.0] vs 65.5 [IQR, 42.5–103.9]; *P* = .025). CRP levels were lower among *Mp*^+^CAP patients (median, 12.9 [IQR, 4.0–22.3] vs 36.7 [IQR, 13.0–132.8]; *P* = .0019), while IP-10 levels were comparable between the 2 groups (median, 366.0 [IQR, 150.2–603.8] vs 331.0 [IQR, 154.3–878.8]; *P* = .73). The distribution of measurements including HCs is shown in Figure 1.

### BV Scores and Co-detection of Pathogens

Referring to co-detections of pathogens in the URT, *Sp* was more frequently detected in *Mp*^+^CAP patients than in *Mp*^−^CAP patients (61.5% vs 27.8%; *P* = .0065). Viral detection rate was low in both *Mp*^+^CAP and *Mp*^−^CAP patients (26.5% vs 9.3%; *P* = .05). When comparing *Mp*^+^CAP patients with and without viral co-detection, we found no statistically significant difference in the BV score (median, 16 [IQR, 7.5–37] vs 8 [IQR, 2.5–25]; *P* = .37). The same was true for BV scores of *Mp*^+^CAP patients with and without *Sp* co-detection in the URT (median, 16 [IQR, 2.5–29.3] vs 8.5 [IQR, 3.3–26.8]; *P* = .88).

### BV Score Categories

With regard to the manufacturer's interpretation categories (ie, score 0–34, suggestive of viral etiology; 35–65, equivocal; and 66–100, indicating bacterial etiology), *Mp*^+^CAP patients yielded a viral score in 21 of 26 (80.8%), an equivocal score in 3 of 26 (11.5%), and a bacterial score in 2 of 26 (7.7%).

### ROC Analysis and Performance Curves

**Figure 2.**
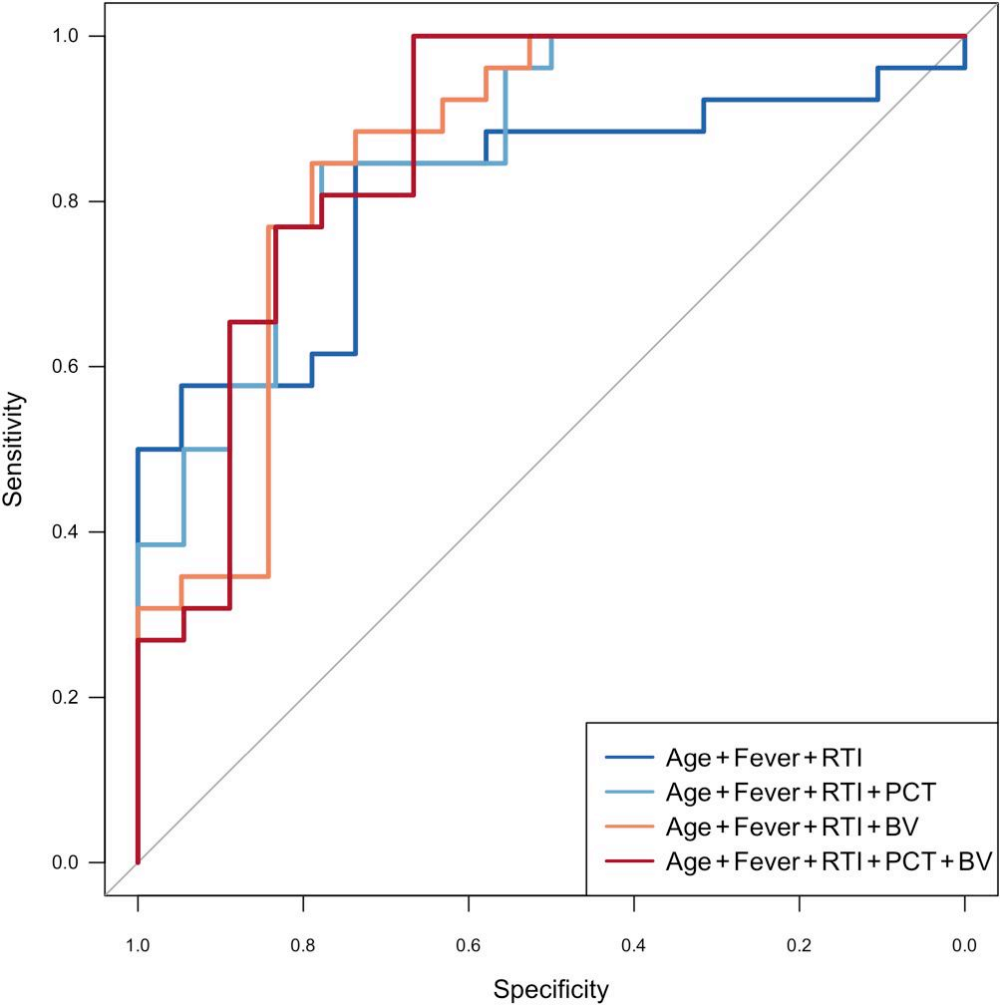
Receiver operating characteristic curves for different combinations of demographic and clinical features and biomarkers. Abbreviations: BV, BV score; fever, duration of fever; PCT, procalcitonin; RTI, duration of respiratory symptoms.

ROC analyses yielded adequate discriminatory accuracies for age (AUC, 0.75), the combination “age + duration of fever + duration of respiratory symptoms” (AUC, 0.81), and the combination “age + duration of fever + duration of respiratory symptoms + PCT” (AUC, 0.87) (Table 2). The combination “age + duration of fever + duration of respiratory symptoms + BV” performed comparable to the combination that included PCT (AUC, 0.86; *P* = .94), while combining the clinical features with both PCT and BV did not result in a significant increase in discriminative potential (AUC, 0.87; *P* = .91) (Figure 2).

**Table 2.** Sensitivity, Specificity, and Area Under the Receiver Operating Characteristic Curve for Different Combinations of Demographic and Clinical Features and Biomarkers

Combination	Sensitivity (95% CI)	Specificity (95% CI)	AUC (95% CI)
Age + duration of fever + duration of respiratory symptoms	0.85 (.69–.96)	0.74 (.53–.89)	0.81 (.68–.94)
Age + duration of fever + duration of respiratory symptoms + PCT	0.85 (.69–.96)	0.78 (.56–.94)	0.87 (.76–.97)
Age + duration of fever + duration of respiratory symptoms + BV score	1.00 (1.00–1.00)	0.67 (.44–.89)	0.86 (.74–.98)
Age + duration of fever + duration of respiratory symptoms + PCT + BV score	0.85 (.69–.96)	0.79 (.63–.95)	0.87 (.75–.99)

Abbreviations: AUC, area under the receiver operating characteristic curve; BV, host-response biomarker score; CI, confidence interval; PCT, procalcitonin.

### BV Scores and *Mp* Load in the URT

**Figure 3.**
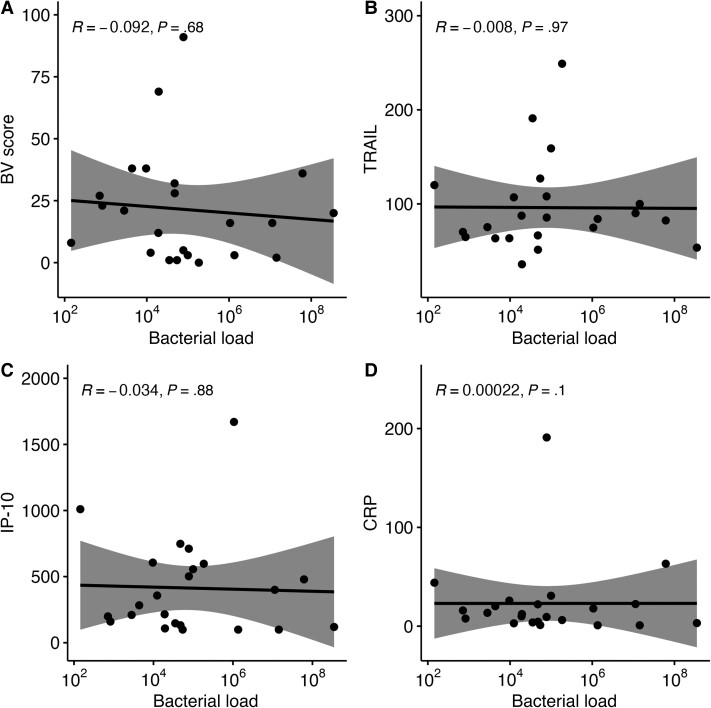
Spearman rank correlation between bacterial load of *Mycoplasma pneumoniae* detected in the upper respiratory tract (x-axes) and the BV score (*A*) and levels of tumor necrosis factor–related apoptosis-inducing ligand (TRAIL; *B*), interferon-γ inducible protein 10 (IP-10, also known as CXCL10; *C*), and C-reactive protein (CRP; *D*) (y-axes). BV indicates the host-response biomarker signature composed of TRAIL, IP-10, and CRP.

We performed correlation analysis for 23 pairs of bacterial load and biomarkers. We found no correlation between bacterial load and BV score, TRAIL, IP-10, or CRP levels (Figure 3).

## DISCUSSION

This study evaluated the host-response biomarker signature “BV score” in *Mp* CAP by using a well-diagnosed cohort, in which *Mp*-specific PCR results were confirmed by the detection of *Mp*-specific IgM ASCs to exclude *Mp* carriers suffering from CAP caused by other pathogens [5]. The study showed that BV scores in *Mp* CAP yielded atypically low levels for “bacterial” infection: The levels in the majority of *Mp* CAP patients were in the range of levels observed during viral infections [1]. We did not find differences in BV scores in those *Mp* CAP patients who were coinfected with a viral pathogen or *Sp* compared to those with *Mp* detection alone. However, the study population was small, and the numbers for specific viruses in particular were too low to draw any conclusions. Testing for *Sp* from the upper airways has been reported to be of limited value. In a randomized controlled trial from the United Kingdom, Little and colleagues found that the presence or absence of pathogens in the upper airways of children with lower respiratory tract infections, including *Sp*, did not predict clinical outcome determined as response to antibiotic treatment [10].

In our study, sampling was performed in a relevant population of clinical CAP and the results confirm our hypothesis that the host-response biomarker signature in *Mp* CAP differs from levels observed during conventional bacterial infections, as previously observed for CRP and other biomarkers [3]. In addition, the differences in the IQRs for the BV score between the 2 groups (narrow in *Mp*^+^CAP, wide in *Mp*^−^CAP) further emphasize that the *Mp* CAP cohort is well-diagnosed, which is reflected by a more uniform BV score expression.

Furthermore, TRAIL levels in *Mp* CAP were significantly higher compared to *Mp*^–^CAP and resembled those found in viral infections, clearly indicating a distinctive immune response in *Mp* CAP [11, 12]. TRAIL has been implicated in the regulation of T-helper cell responses [13]. We previously showed that host cell-mediated immunity, particularly pathogen-specific T-cell responses, contributes to *Mp* pulmonary disease [14]. Interestingly, TRAIL has been shown to increase the effects of respiratory infection with other atypical bacteria, such as *Chlamydia* spp, suggesting that TRAIL may enhance infection-mediated immunopathology [15]. However, further studies are required to reveal the exact role of TRAIL in *Mp* pulmonary disease.

The results of our analyses are especially relevant to clinicians who, when using novel biomarkers as those included in the BV score, could misdiagnose *Mp* CAP as viral infection. Although *Mp* infections are mostly mild, self-limiting, and managed in primary care, severe disease and extrapulmonary manifestations may occur [3]. It is therefore important to correctly diagnose and adequately treat such complications. The detection of specific ASCs by ELISpot assay could help in these cases by differentiating between *Mp* infection and carriage [5, 6].

A limitation of the ASC ELISpot assay as well as of the BV score (yet) is the requirement of venous blood, which will not be obtained in most children with *Mp* CAP managed in primary care. Using the ASC ELISpot assay, we were able to determine clinical features that may aid physicians in identifying patients at high risk for *Mp* CAP [3]. We showed that the diagnostic performance of these clinical features could be further improved by combining with results of the BV score. Thus, when the BV score becomes available for capillary whole blood, the combination of clinical features and biomarkers included in the BV score may even better help physicians in guiding diagnosis and treatment in the outpatient setting. Optimizing protocols to allow a more rapid (∼6–8 hours vs ∼24 hours) ASC detection will also help to routinely use the *M*p-specific ASC ELISpot assay for diagnosing *Mp* CAP [6].

## CONCLUSIONS

Our findings show that the BV score and its subanalytes have limitations for atypical bacteria, which underscores the complementary role of microbiological testing to guide targeted antibacterial treatment. However, identifying the etiology of CAP is challenging, and microbiological testing is generally recommended to attempt an etiological diagnosis of CAP in patients requiring hospitalization.

## Supplementary Data

Supplementary materials are available at *The Journal of Infectious Diseases* online (http://jid.oxfordjournals.org/). Supplementary materials consist of data provided by the author that are published to benefit the reader. The posted materials are not copyedited. The contents of all supplementary data are the sole responsibility of the authors. Questions or messages regarding errors should be addressed to the author.

## Supplementary Material

jiad573_Supplementary_Data

## Notes

***Author contributions.*** Conceptualization and supervision: C. P. and P. M. M. S. Funding: C. P. and P. M. M. S. Data generation and curation: All authors. Data analysis: C. P. and P. M. M. S. Data interpretation: All authors. Writing: C. P. and P. M. M. S. Review and agreement with the final version of the manuscript: All authors.

***Acknowledgments.*** The authors thank all children and their parents who contributed to this study and the staff involved in the study.

***Ethics statement.*** The study was approved by the local ethics committee (reference number 2016-00148).

***Financial support.*** Funding to pay the Open Access publication charges for this article was provided by Saarland University and University of Zurich. DiaSorin kindly provided cartridges for BV measurements. DiaSorin had no role in the conception, design, interpretation, reporting, and publication of the study.
