# Supplementary material for: Combinatorial Host-Response Biomarker Signature (BV Score) and Its Subanalytes TRAIL, IP-10, and C-Reactive Protein in Children With Mycoplasma pneumoniae Community-Acquired Pneumonia
Source: J Infect Dis. 2023 Dec 13;230(2):e247–53. doi: 10.1093/infdis/jiad573 (PMC11326813; doi:10.1093/infdis/jiad573)
Supplement: jiad573_Supplementary_Data [file jiad573_supplementary_data.docx]

**SUPPLEMENTARY APPENDIX**

Supplement to:
Papan C, Sidorov S, Greiter B, et al.

Combinatorial host-response biomarker signature (BV score) and its subanalytes TRAIL, IP-10, and CRP in children with *Mycoplasma pneumoniae* community-acquired pneumonia

**Supplementary Table 1.** Comparison between the study cohort and the previously published cohort of pediatric CAP patients and healthy controls (HCs).

| *Mp* test result | **Study cohort with additional serum samples available for testing with the Liaison MeMed BV** | | Previously published cohort [1] | |
| --- | --- | --- | --- | --- |
|  | **CAP**  **(*n*=80)** | **HC**  **(*n*=25)** | CAP  (*n*=152) | HC  (*n*=156) |
| PCR-positive* | **26 (33%)** | **5 (20%)** | 44 (29%) | 12 (8%) |
| *- IgM ASC ELISpot-positive* | *26/26 (100%)* | *0/5 (0%)* | *29/32 (91%)* | *0/10 (0%)* |
| *- IgM ASC ELISpot-negative* | *0/26 (0%)* | *5/5 (100%)* | *3/32 (9%)* | *10/10 (100%)* |
| PCR-negative* | **54 (68%)** | **20 (80%)** | 108 (71%) | 144 (92%) |
| *- IgM ASC ELISpot-positive* | *0/27 (0%)* | *0/11 (0%)* | *0/31 (0%)* | *0/11 (0%)* |
| *- IgM ASC ELISpot-negative* | *27/27 (0%)* | *11/11 (100%)* | *31/31 (100%)* | *11/11 (100%)* |

*Not all patients tested with the *Mp*-specific PCR did have fresh (isolated ≤4 hours) peripheral blood mononuclear cells (PBMCs) available for additional testing with the *Mp*-specific ASC ELISpot assay, as previously reported [1].

1. Meyer Sauteur, P.M., Seiler, M., Truck, J., Unger, W.W.J., Paioni, P., Relly, C., et al. **Diagnosis of Mycoplasma pneumoniae Pneumonia with Measurement of Specific Antibody-Secreting Cells.** *Am J Respir Crit Care Med*. 2019; **200**: 1066-1069
